# Supplementary figures and images for: Numerical evaluation reveals the effect of branching morphology on vessel transport properties during angiogenesis
Source: PLoS Comput Biol. 2021 Jun 16;17(6):e1008398. doi: 10.1371/journal.pcbi.1008398 (PMC8238234; doi:10.1371/journal.pcbi.1008398)

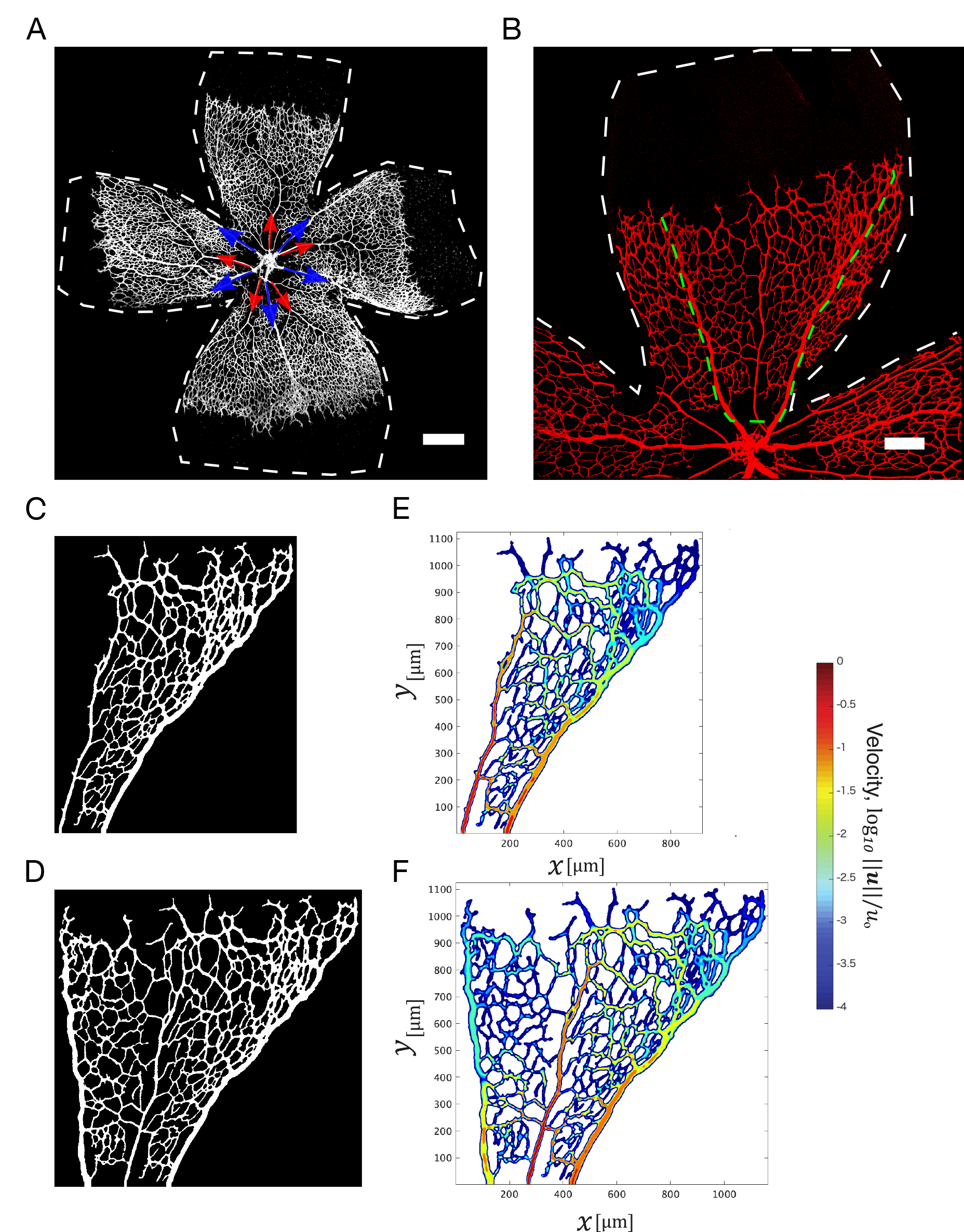

Supplement: S1 Fig — A) Staining of ICAM-II in a wild-type mouse retina at P5. White dashed line indicates the retinal tissue border. Red and blue arrows show the retinal radiating arteries and veins, respectively; Scale bar represents 500 μm. B) Staining of ICAM-II in one lobe of a wild-type P5 retina from which the A-V and V-A-V structures were extracted. White and green dashed lines indicate the tissue border and isolated V-A-V region, respectively; Scale bar represents 200 μm. C) Black and white A-V and D) V-A-V structures derived from the same vascular bed (B) for flow simulation. E) Visualization of the velocity amplitude on the central x-y plane (along the z axis) for the A-V and F) the V-A-V structures; Color scale represents the logarithmic form of the normalized velocity (log10‖u‖/uo), where uo is the outlet velocity in the right vein. (TIF) [file pcbi.1008398.s001.tif]

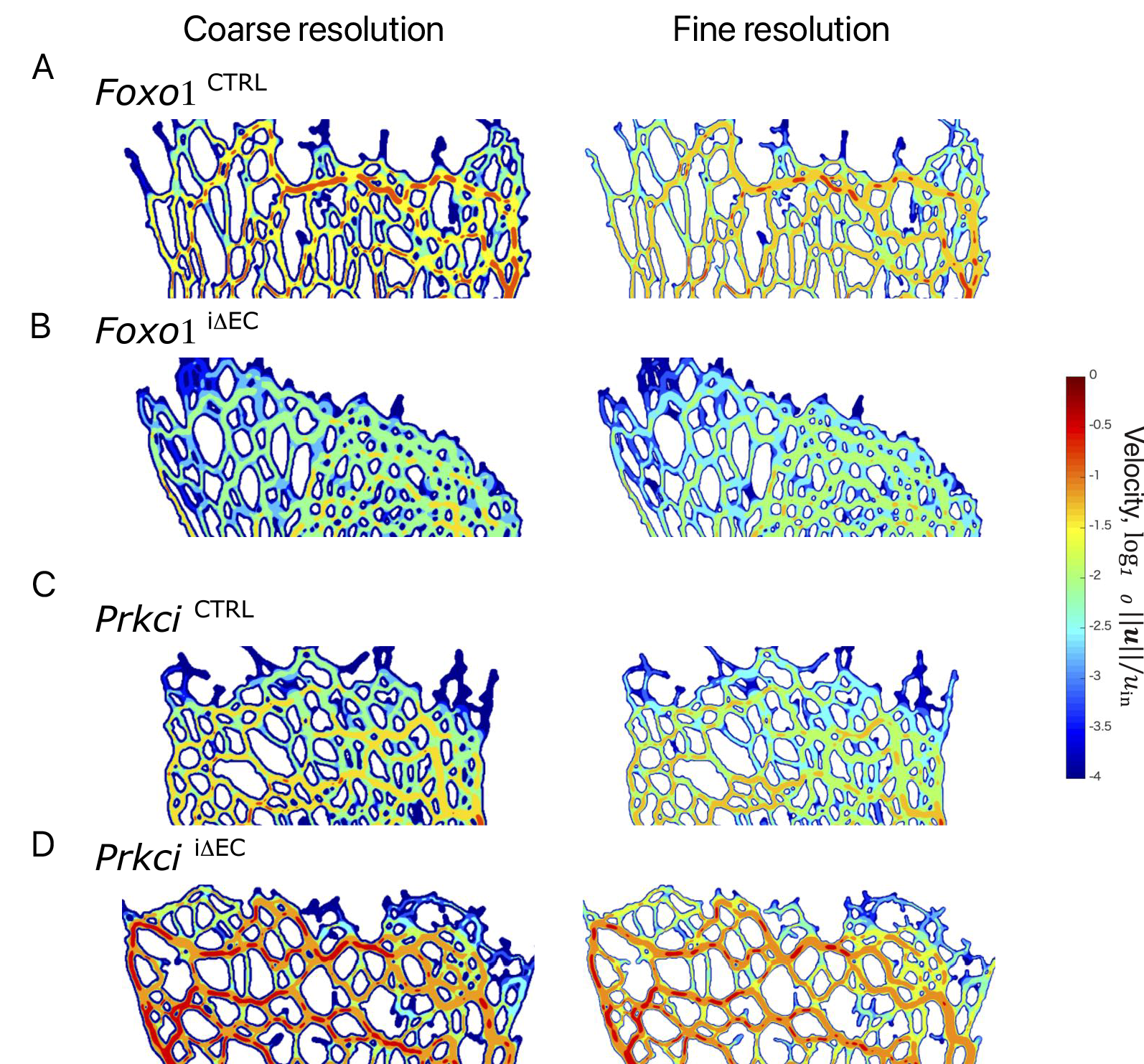

Supplement: S2 Fig — A) Foxo1 CTRL, B) Foxo1 iΔEC, C) Prkci CTRL, D) Prkci iΔEC; Color scale represents the logarithmic form of the normalized velocity (log10‖u‖/uin). (TIF) [file pcbi.1008398.s002.tif]

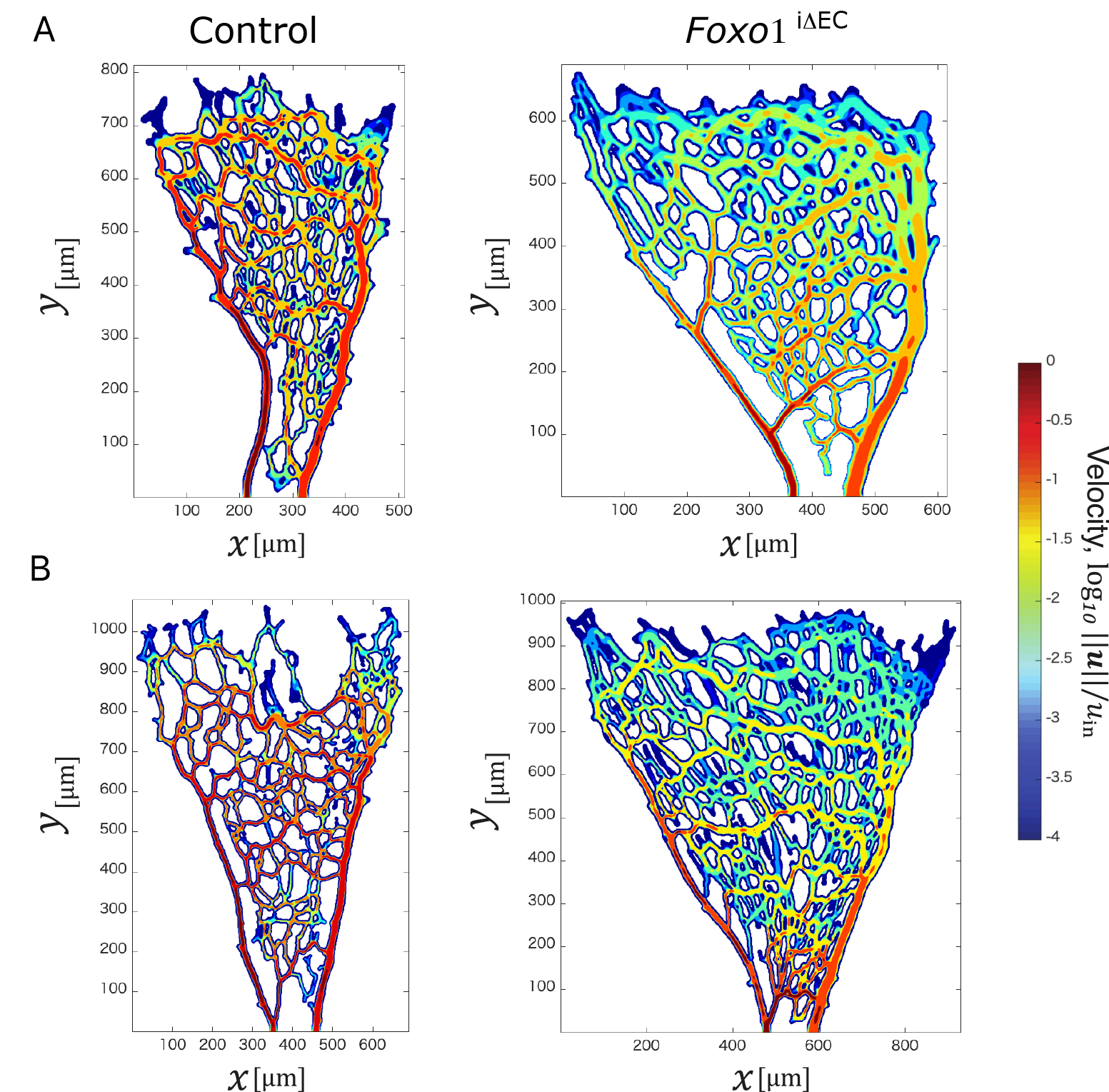

Supplement: S3 Fig — A) Visualization of the normalized amplitude on the central x-y plane (along the z axis) for the second and B) the third sets of control and Foxo1iΔEC retinas each from different litters; Color scale represents the logarithmic form of the normalized velocity (log10‖u‖/uin). (TIF) [file pcbi.1008398.s003.tif]

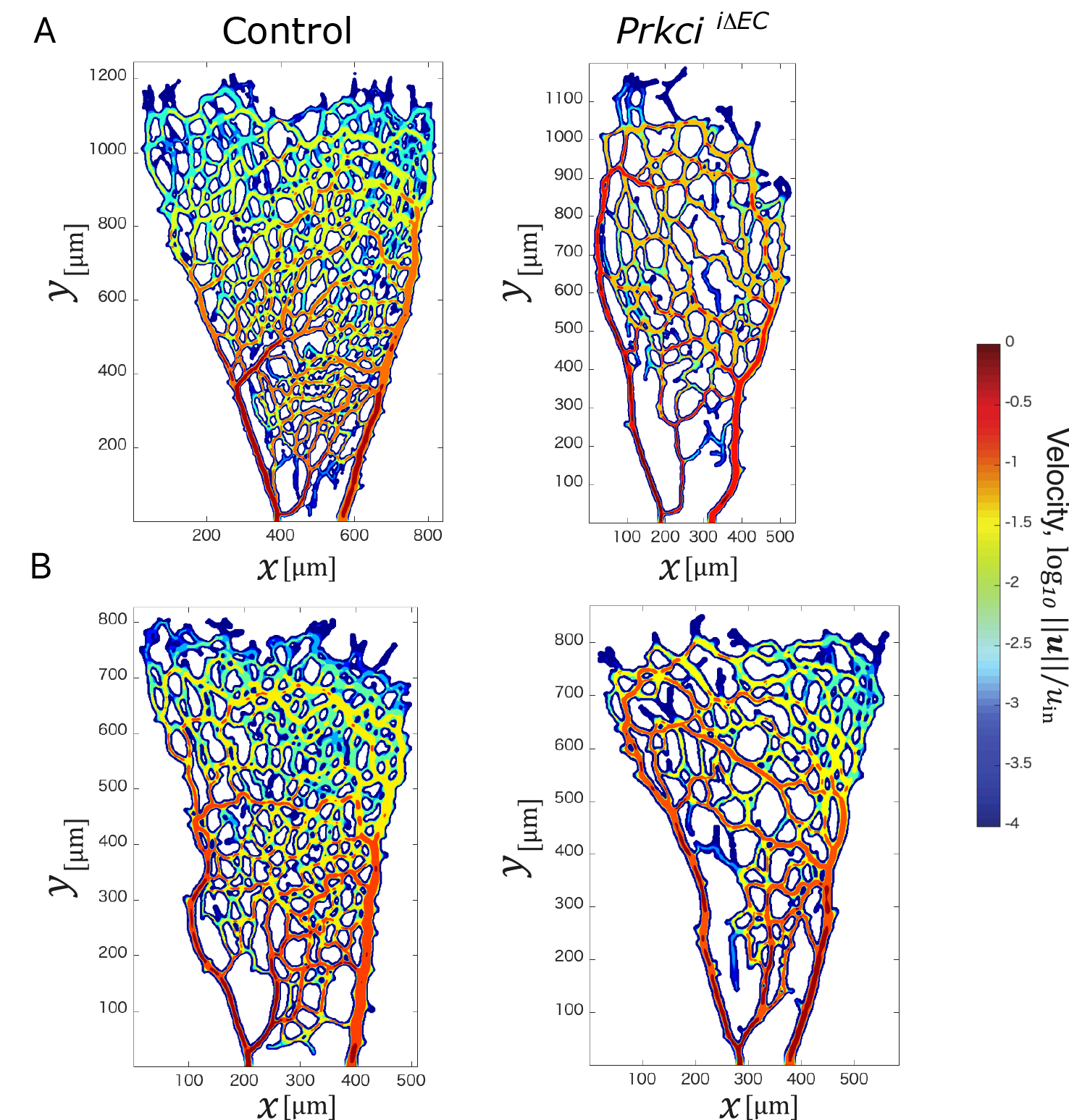

Supplement: S4 Fig — A) Visualization of the velocity amplitude on the central x-y plane (along the z axis) for the second and B) the third sets of Control and PrkciiΔEC retinas each from different litters; Color scale represents the logarithmic form of the normalized velocity (log10‖u‖/uin). (TIF) [file pcbi.1008398.s004.tif]

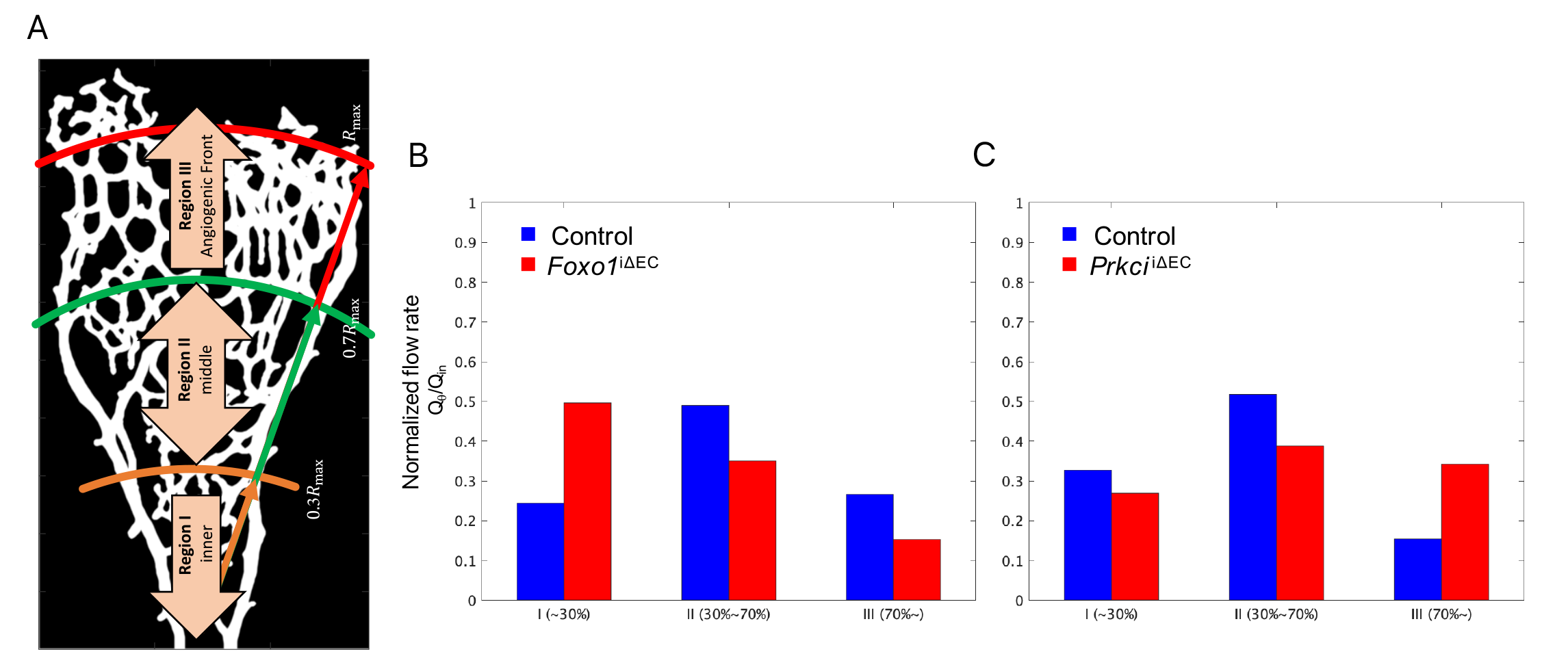

Supplement: S5 Fig — A) Decomposition of the entire vascular network into three regions, i.e., I: inner region (0–30%), II: middle region (30–70%), III: outer region (70–100%). B) The averaged azimuthal flow rates at the three regions for the Foxo1 and C) Prkci mutants and the respective controls at P5. In (B) and (C), the red and blue bars represent the results of mutant and control cases, respectively. (TIF) [file pcbi.1008398.s005.tif]

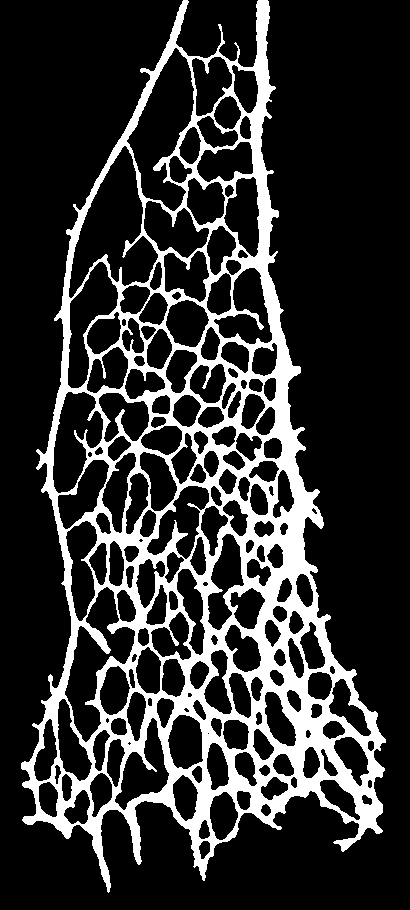

Supplement: S1 Data — (GZ) [file pcbi.1008398.s007.gz › Mirzapourshafiyi_etal_2021_SampleCodes/binarization/Sample_vMask_Vertical.tif]

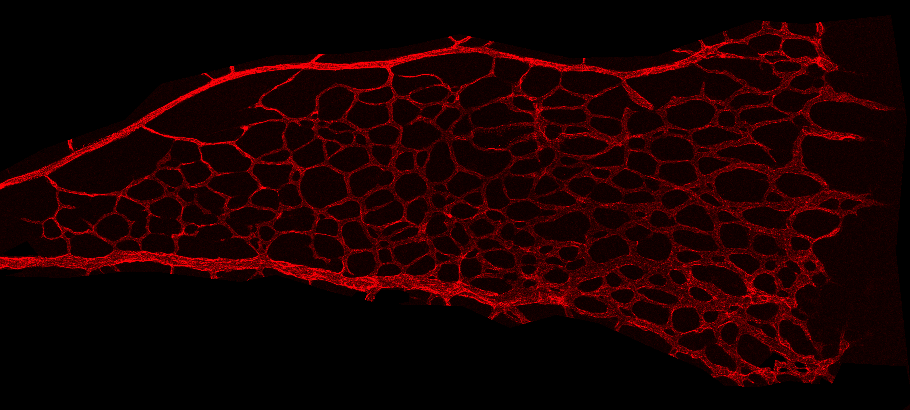

Supplement: S1 Data — (GZ) [file pcbi.1008398.s007.gz › Mirzapourshafiyi_etal_2021_SampleCodes/binarization/Sample_Unit.tif]

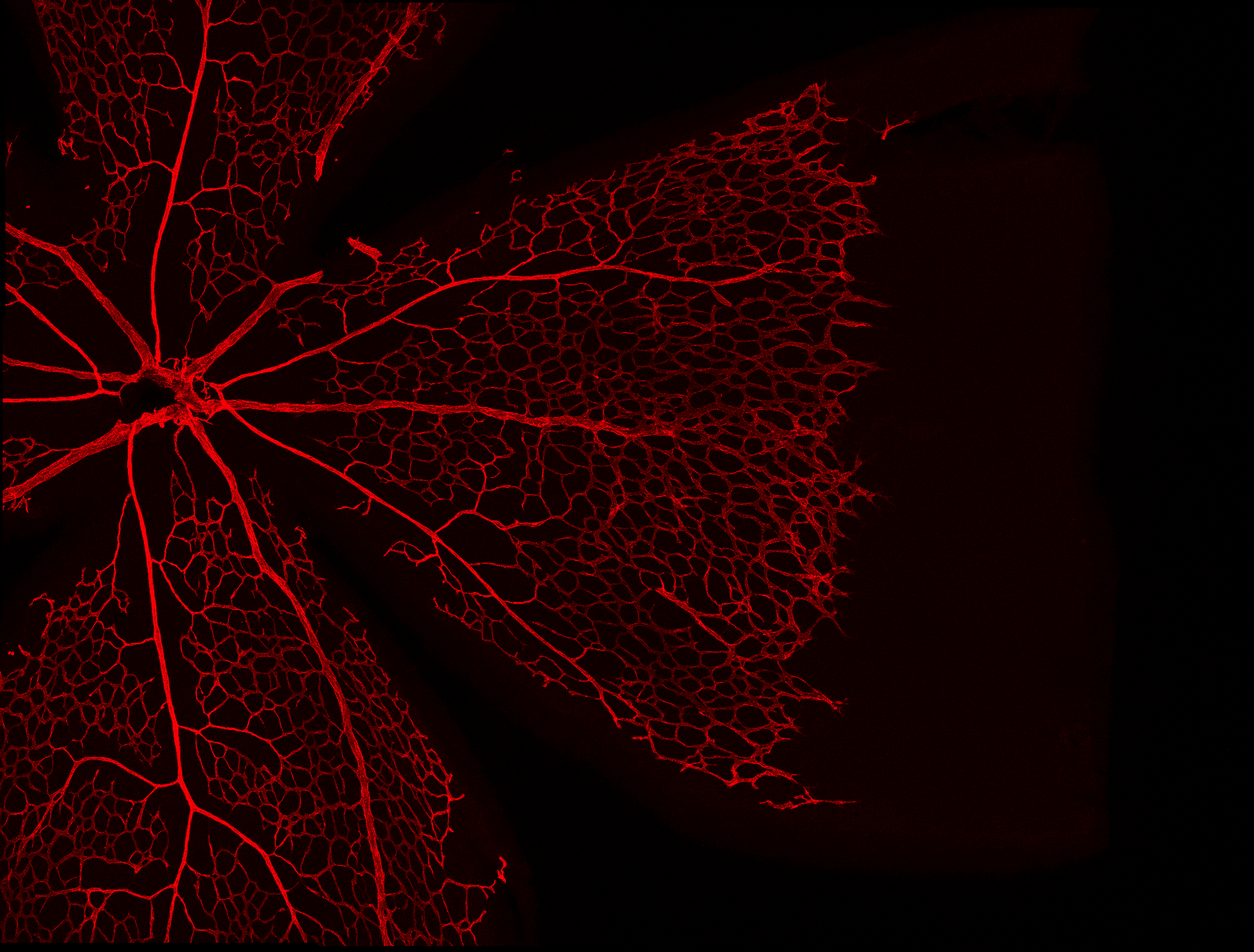

Supplement: S1 Data — (GZ) [file pcbi.1008398.s007.gz › Mirzapourshafiyi_etal_2021_SampleCodes/binarization/Sample_Image.tif]
