# Supplementary material for: Numerical evaluation reveals the effect of branching morphology on vessel transport properties during angiogenesis
Source: PLoS Comput Biol. 2021 Jun 16;17(6):e1008398. doi: 10.1371/journal.pcbi.1008398 (PMC8238234; doi:10.1371/journal.pcbi.1008398)
Supplement: S1 Table — (DOCX) [file pcbi.1008398.s006.docx]

|  | Coarse resolution | | Fine resolution | |
| --- | --- | --- | --- | --- |
|  | Inner Plexus | Angiogenic front | Inner Plexus | Angiogenic front |
| *Foxo1* CTRL | 75.0 % | 25.0 % | 77.8 % | 22.2 % |
| *Foxo1* ^iΔEC^ | 84.2 % | 15.8 % | 87.6 % | 12.4 % |
| *Prkci* CTRL | 78.7 % | 21.3 % | 81.8 % | 18.2 % |
| *Prkci* ^iΔEC^ | 62.9 % | 37.1 % | 65.7 % | 34.3 % |
