# Supplementary material for: Numerical evaluation reveals the effect of branching morphology on vessel transport properties during angiogenesis
Source: PLoS Comput Biol. 2021 Jun 16;17(6):e1008398. doi: 10.1371/journal.pcbi.1008398 (PMC8238234; doi:10.1371/journal.pcbi.1008398)
Supplement: S1 Text — (DOCX) [file pcbi.1008398.s008.docx]

**S1 Text**

**Validity of considering a minimum flow unit**

As discussed in Sec. 2.3., the current study considers a minimum flow unit between an artery and a vein (‘A-V region’). This choice has been made due to several experimental and numerical limitations:

First, due to the hemispherical geometry of the retinal tissue, radial cuts are needed in order to facilitate the flat mounting of the tissue for imaging (see S1A Fig). With the four cuts dividing the retinal tissue into four flat lobes, the retinal vascular network is damaged. Also, some parts of the retinal tissue would be damaged during the staining processes. Consequently, it was difficult to find a whole region containing intact connections of artery, vein and all the capillaries, from the ONH to the angiogenic front.

Second, numerical setup becomes complicated when there exist multiple inlets and outlets. In addition, the computational cost becomes large in proportion to the size of the computational domain.

Strictly speaking, however, each flow unit is not completely independent, but coupled with neighboring units. Specifically, the blood injected from one artery should be distributed to both sides of the capillary bed and eventually flows into two neighboring veins. In order to clarify how such coupling between neighboring units affects the current results, the results of single unit (A-V region) and double unit (V-A-V region) are compared in S1 Fig. Note that the velocity contours shown in S1E and S1F Fig are both normalized by the outlet velocity in the right vein, which corresponds to the total flow rate on the right side of the middle artery. It can be confirmed that the coupling between neighboring units hardly affects the flow distribution when compared with the results obtained for a single flow unit. Specifically, the normalized azimuthal flow rate in the angiogenic front is changed by less than 5% when V-A-V region is considered. This value is sufficiently smaller than those caused by gene knockout, so that it will not alter the present conclusions.
