# Supplementary material for: Numerical evaluation reveals the effect of branching morphology on vessel transport properties during angiogenesis
Source: PLoS Comput Biol. 2021 Jun 16;17(6):e1008398. doi: 10.1371/journal.pcbi.1008398 (PMC8238234; doi:10.1371/journal.pcbi.1008398)
Supplement: S2 Text — (DOCX) [file pcbi.1008398.s009.docx]

**S2 Text**

**Grid convergence study**

To verify the current grid resolution, additional numerical simulations with doubled grid resolutions in all the directions were performed. Due to the large computational cost, we considered one set of KO mice and their control littermates (CTRL) for each of the *Foxo1* and *Prkci* cases. They are denoted as *Foxo1* CTRL-1F, *Foxo1 ^i^*^∆EC^-1F, *Prkci* CTRL-1F and *Prkci ^i^*^∆EC^-1F, respectively. The numbers of grid points employed for the simulations with finer grid size are listed in the bottom of Table1.

The distributions of the velocity intensity around the angiogenic front obtained with current and finer grid resolutions are compared in S2 Fig. The left column contains the results obtained with the initially coarse resolution, whereas the right column shows those with the finer resolution. In general, the overall trend does not change with increasing the grid resolution. Specifically, it can be confirmed that the flow rate decreases at the angiogenic front region of the *Foxo1^i^*^∆EC^ models, whilst it is enhanced in the same region of the *Prkci*^iΔEC^ structures in comparison to the corresponding control structures. More quantitatively, S1 Table summarizes the average flow portion (integrated flow rate in percentage) in the inner plexus and angiogenic front regions of the *Foxo1^i^*^∆EC^ and *Prkci*^iΔEC^ cases and their controls, before and after grid refinement. In general, the simulation with the coarser grids tends to overestimate the averaged flow portion in the angiogenic region by around 3%. This could be explained by better prediction of flow distributions within small-diameter branching vessels in the inner plexus with the grid refinement. The decrease of the averaged flow rate in the angiogenic region due to *Foxo1* knockout is estimated as 9.2% for the coarse resolution, whereas 9.8% for the finer one. Similarly, knocking out *Prkci*^iΔEC^ increases the flow rate in the angiogenic region by 15.8% and 16.1% for the coarse and fine resolutions, respectively. Hence, it can be confirmed that further grid refinement from the initial coarse resolution has only minor impacts on the current results, and does not affect the current conclusions.
