# Supplementary material for: Numerical evaluation reveals the effect of branching morphology on vessel transport properties during angiogenesis
Source: PLoS Comput Biol. 2021 Jun 16;17(6):e1008398. doi: 10.1371/journal.pcbi.1008398 (PMC8238234; doi:10.1371/journal.pcbi.1008398)
Supplement: S3 Text — (DOCX) [file pcbi.1008398.s010.docx]

**S3 text**

**Effects of morphological changes to flow rates at different regions**

As explained in Sec 3., in the present study, the entire vascular bed is divided into the inner (0-70%) and outer (70-100 %) regions, and the azimuthal flow rate in the outer region is calculated in order to evaluate the transport efficiency of the vascular networks. However, the presented results do not fully answer the question specifically which region has a larger impact on the azimuthal flow rate in the angiogenic front. Hence, we further divide the inner region into two regions, so that the entire vasculature is now divided into three regions, i.e., inner (0-30%), middle (30-70%) and outer (70-100%) regions as shown in S5A Fig.

The plots shown in S5B Fig and S5C Fig show the changes in the azimuthal flow rates within the three regions for the network from *Foxo1* and *Prkci* KO mice. The red and blue bars represent mutant and control cases, respectively. As already discussed in Sec. 3, the flow rate in the outer region decreases for *Foxo1* KO (hype-branching) and increases for *Prkci* KO (hypo-branching) networks, respectively. Since we divided the original inner region into two, more detailed information can be obtained. Specifically, in the case of *Foxo1* KO mice, even though the network evenly becomes dense within the entire vasculature, the flow rate is selectively increased in the immediate inner region (0-30%), while the flow rate already starts decreasing in the middle region (30-70%). This indicates that the inner shunt close to the central region has a significant impact on the downstream flow distribution. In contrast, in the case of *Prkci* KO, the flow rates in both the inner and middle regions are reduced due to hypo-branching, and the corresponding increase in the flow rate occurs in the outer region. It would be interesting to divide the entire vasculature into further narrower regions for more detailed investigation of local contributions, but it increases variations due to the lack of the number of samples.
