# Supplementary material for: Numerical evaluation reveals the effect of branching morphology on vessel transport properties during angiogenesis
Source: PLoS Comput Biol. 2021 Jun 16;17(6):e1008398. doi: 10.1371/journal.pcbi.1008398 (PMC8238234; doi:10.1371/journal.pcbi.1008398)
Supplement: Attachment — Submitted filename: PLOSrevision_letter_0201MNYHTH.docx [file pcbi.1008398.s011.docx]

We are very grateful to all of the reviewers for their positive evaluation of the paper, and would like to thank them for their constructive feedback working towards improving our manuscript. As you will see, we have extensively revised the manuscript. A detailed point-by-point response to all comments is given below. Corresponding revisions are highlighted by red in the revised manuscript. We believe that our revised manuscript indicates the effect of branching morphology on transport property of the vascular morphology and thereby highlining an importance of our novel approach to evaluate blood vessel formation and function.

**[Comments from Reviewer1]**

**Reviewer #1**: This manuscript supplies a simulation of blood flow in a murine retinal vascular network obtained from microscopy images, to determine how vascular branching affects blood flow in angiogenesis. Interestingly, results indicate that hyperbranching in the network leads to reduced flow, and hypobranching leads to increased flow, during angiogenesis. This simulation result is implicitly supported by experimental results indicating increased VEGF expression due to lower oxygenation in the angiogenic front of the hyperbranching vasculature.

Minor issues:

Comment 1-1: It would be nice to see a paragraph in the Introduction and possibly also in the Discussion about other studies simulating blood flow (and possibly oxygenation) in the microcirculation -- what exactly has been done before? On line 73-74, the manuscript says "the effects of altered vascular branching on blood flow distribution and the oxygen transport property have not been investigated yet", but there are plenty of modeling studies that attempt to predict blood flow in a branching network. It would be nice to have some more details about how these are being improved upon in this study, at least mentioning that this study may be the first to use this particular realistic mouse retinal network during angiogenesis.

[Answer]

As the reviewer pointed out, there exist numerous attempts to simulate blood flow and associated oxygen transport. However, there exist no study systematically comparing transport properties of vascular networks with different morphological features. This might be achieved by artificially removing/adding vessels from/to a reference structure based on statistical or empirical rules (T. Köppl et al. 2020, T. W. Secomb et al. 2013). However, the resultant structures are no longer real, so that no experimental validation is possible.

The main features in the present study are to apply genetic modification techniques to realize hyper- and hypo-branching vascular network in mouse retina and to systematically analyze blood flow and associated oxygen transport for the real structures with different morphological features. This allows us to compare numerical results with experimental observation for validation.

The above points have clearly been addressed in the second and third paragraph of Sec. 1 (Introduction) of the revised manuscript.

[ref]

- T. Köppl et al. “3D-1D coupled blood flow and oxygen transport model to generate microvascular networks” Int J Numer Method Biomed Eng. 2020.
- T. W. Secomb et al. “Angiogenesis: An Adaptive Dynamic Biological Patterning Problem” PLoS Comput Biol., 2013.

Comment 1-2: In the first paragraph of the "Numerical simulation of blood flow" section (line 115), it is explained why a Newtonian assumption is made about the blood flow; however, in the Discussion, starting with line 241, there appears to be an argument for why flows in the microcirculation cannot be represented in this way. It would be good to have a reminder in the Discussion as to why it's acceptable to assume constant viscosity in this case, and/or describe the limitations of this assumption on the model predictions.

[Answer] The Newtonian assumption is considered reasonable, at least, within a single vessel according to existing literatures as discussed in the section "Numerical simulation of blood flow". However, these evidences have been obtained in relatively straight vessels sufficiently away from branching points (e.g., Logean et al. 2003). In contrast, near a branching point, the effects of three-dimensionality and axial flow development could be important. However, these effects are neglected in 1D analyses (Sherwin et al. 2003, Formaggia et al., 2003). It is also known that red blood cells are not evenly distributed from a parent vessel to daughter vessels. This so-called skimming effect originally pointed out by Krogh (1921) reduces the hematocrit value of a smaller vessel from that of large artery and vein. Therefore, even though the Newtonian assumption is reasonably well for each branch, the effective viscosity could be gradually changed depending on the generation of a branch from the artery or vein. The above results suggest that 3D effects are important to analyze the blood flow and resultant transport properties in capillary network. Although our current study does not explicitly take the dynamics of red blood cells and their interaction with plasma flow into account, the present framework can be extended for such purposes in future work. We addressed these points clearly in the second paragraph of Sec. 5 (Discussion) in the revised manuscript.

[ref]

- E. Logean, L. Schmetterer, C.E. Riva “Velocity profile of red blood cells in human retinal vessels using confocal scanning laser doppler velocimetry” Laser Physics, Vol. 13, No.1, 45-51, 2003.
- S. Sherwin, V. Franke, J. Peiro, K. Parker “One-dimensional modelling of a vascular network in space-time variables”, J. Eng. Math. Vol. 47, No.3-4, 217-250 (2003)
- L. Formaggia, D. Lamponi, A. Quarteroni, “One-dimensional models for blood flow in arteries”, J. Eng. Math. Vol. 47 No. 3-4, 251–276 (2003).
- A. Krogh "Studies on the physiology of capillaries II. The reaction to local stimuli of the blood-vessles in the skin and web of the frog" The Journal of Physiology Vol. 55, Issue 5-6, pp.412-422 1921.

Comment 1-3: The ordering of section titles is a bit difficult to follow; it seems to go Introduction, Results, Discussion, Materials and Methods -- although part of the Results section seems to include some Methods. I would suggest moving Materials and Methods to after the Introduction, and possibly even include all of the following subsections: "Image Acquisition", "3D reconstruction", and “Numerical simulation", since these all appear to describe Methods. Then have "Simulation results" and "Experimental validation” as subsections of a Results section.

[Answer] Following the reviewer’s comment, we insert a new section (Sec. 2 in the revised manuscript) summarizing methods used in experiment and simulation after Sec. 1 (Introduction) in the revised manuscript. As a result, the structure of the manuscript has been revised as

1. Introduction
2. Materials and Methods
3. Simulation Results
4. Experimental Validation
5. Discussion
6. Supplementary Information

Very minor things:

Comment 1-4: * Page 6, line 134: “define" should be "defined"

[Answer] Thank you. Now it is corrected in the revised manuscript.

Comment 1-5: * Page 7, line 136: I think there should be a comma after the word "time-scale"

[Answer] The original sentence was long and not easy to read, so that we revised the sentence as follows “This dimensionless number is defined as the ratio between the time-scale for the wall information propagating to the bulk fluid via fluid viscosity and the pulsation period.” (Line 185 in the revised manuscript)

Comment 1-6: * Page 7, line 138: "pulsations" should be "pulsation's"

[Answer] Thank you for the correction. After reading the sentence, we realized that it was somewhat redundant since a similar content was already written in a previous sentence. Therefore, we removed the entire sentence from the revised manuscript.

Comment 1-7: * Page 9, line 202: The line "Here, Rmax is the distance of the farthest blood vessel from the origin" can be removed, since it appears just above, in lines 199-200.

[Answer] The above redundant sentence has been removed from the revised manuscript.

**[Comments from Reviewer2]**

**Reviewer #2**: Review of "Numerical evaluation reveals the effect of branching morphology on vessel transport properties during angiogenesis" by Mirzapour-shafiyi et al.;

In summary - I think this is an interesting study, but I don't think the interpretation/claims are yet supported by the evidence provided. I would be interested to see a revised submission addressing these critiques.

Comment 2-1: Because all tissue vascular beds are different, it took me a little while to work out why u_theta is being used as the relevant velocity for delivery of blood flow to the periphery rather than u_r, and if I am correct I think the paper would benefit from further description. I think it is as follows: the simulated tissue wedge includes both an artery and a vein (the two large radial vessels one ither side of the wedge); since flow should go from artery to vein, u_theta as the flow "around the circle" measures the 'direct' route from artery to vein via peripheral tissue. I don't think that's explained in the manuscript. Fig 2D and 3A note the artery/vein pair, but the choice of flow unit and interpretation is not really described, as far as I can tell.

[Answer] Thank you for the comment. As correctly pointed out by the reviewer, the simulated vascular bed of the tissue wedge includes a pair of radial branches of one feeding artery and one draining vein at either side. To quantify the amount of flow transported from the artery to the vein through the capillary network, we introduced a cylindrical coordinate system (r -θ) and decomposed the local flow velocity vector into the radial and azimuthal components, i.e., ur and uθ. As the azimuthal component of the velocity, uθ, measures the direct flow route from the artery to the vein through the microvascular bed. Therefore, integration of uθ would calculate the transported flow rate from the artery to the vein across the region. This description has been added to the second paragraph of Sec. 3 (Simulation Results) by referring to a newly inserted figure S1 in the revised manuscript.

Comment 2-2: But also, let's unpack that: the retinal structure is alternating A-V-A-V around the circle. The flow in an artery does not go only to one vein; there is another vein on the other side to which (presumably) half the flow would go. I don' t think it will substantially change the findings here, given the normalization being done for the analysis, but the A-V wedge as chosen is not really a full flow unit.

[Answer] The reviewer is completely right. In the present study, due to experimental limitations and also computational cost, only a minimum flow unit, i.e., a A-V unit, was chosen for the current study. In order to validate the current approach, we performed additional simulations for A-V and V-A-V units extracted from the same P5 retinal vascular bed. Here, in the new V-A-V configuration, the middle artery feeds to two veins on both sides. It was confirmed that the resulting simulated flow velocity distribution (supplemental Fig. S1) did not show any substantial difference between the two configurations. We assessed the normalized azimuthal flow rate (*Q_θ_*) in the same A-V section of both the A-V and V-A-V structures, to measure the portion of the flow transported to the angiogenic front. The difference in the calculated *Q_θ_* for the same A-V section of the A-V and V-A-V structures was found to be very minor comparing to the *Q_θ_* difference between the control and KO populations in both of the hyper- and hypo-branching models. These results have been added in Supporting Information Sec 6.1, and it is properly referred in the first paragraph of Sec. 2.3 of the revised manuscript.

Comment 2-3: Given the difference between ||u|| and u_theta, we are left with a question as to what u_r represents. Might it be a metric of heterogeneity of flow delivery to the front (i.e. a metric of how much local branches have to compensate for unbalanced distribution - though one would have to take care with sign of velocity when integrating), or maybe a more 'bulk' interpretation where if azimuthal blood flow is low, radial flow would be higher to try to supply that region from inner regions? It might depend on whether we're looking at u_r in the peripheral, inner, or boundary regions. Note also that u_r is not graphed - perhaps it is too similar to ||u||? If so, this should be stated.

[Answer] Thank you for the comment. Indeed, u_r is also an interesting quantity to look at. Meanwhile, the blood is assumed to be incompressible in the present study. Therefore, the azimuthal flow from the artery to the vein has to be exactly compensated by the decrease of the radial velocity so as to satisfy mass conservation. In this sense, there is one-to-one relationship between u_r and u_theta. This is a main reason why we did not show the plot of u_r.

Just for reference, we include the distribution of u_r in this reply (see, Fig. R1 below). As the reviewer pointed out, the distribution of u_r is indeed quite similar to that of ||u|| shown in the original manuscript, and it decreases with increasing the distance from the inlet. Therefore, we decided not to include the plot of u_r in our manuscript. Instead, the above explanations have been added by texts at second to the last paragraph of Sec. 3 in the revised manuscript.

Figure R1. Distributions of (left) absolute, (middle) azimuthal and (right) radial velocity components.

Comment 2-4: Also, can you explain why the normalized azimuthal flow rate is substantially different between the two control populations (Fig 3G vs 3H)?

[Answer] Thank you for pointing this out. Using two different mouse lines to obtain the hyper- and hypo-branching phenotypes causes variations in litter and animal sizes. During retinal angiogenesis from postnatal day 0 (P0) to P7, the blood vessel expands rapidly and extensively on the surface of retina. In other words, the vasculature dramatically grows even in a couple of hours, which could cause huge variations in vascular morphology across different mouse lines even in wild type animals.

Due to the above reasons, meaningful conclusions can be drawn only through comparisons between KO and control animals within littermates. This is a reason why we compare KO and WT within the same mouse line. These explanations have been added in the last paragraph of Sec. 3 (Simulation Results).

Comment 2-5: The modeling engine itself is reasonably well explained; but some questions remain about the choice of modeling approach, including: does the 3d analysis improve on 1d flow analysis? A 1d analysis should be able to provide u_r and u_theta values (and Q values) based on the orientation and diameters of the vessel segments, so the comparison should be made given that this manuscript is introducing an alternate approach and claiming that it is superior.

[Answer] The comparison between 1D and 3D analyses are indeed interesting. Although 1D analysis is much cheaper than 3D analysis, the image preprocessing for 1D analysis is not an easy task. For example, in order to perform 1D analysis of the present configuration, the center line and the local diameter of each vessel and also the connectivity between neighboring vessels should be accurately extracted from a confocal image. It is relatively easy for a small subset of vessels, but it requires significant trials-and-errors for a large domain considered in the present study. Therefore, we currently focus on a much smaller domain for the detailed comparison between 1D and 3D analyses, and it will be discussed in another publication. Considering the above cumbersome preparation required in 1D analysis, reconstructing a 3D structure from a confocal image and directly integrating it to 3D flow simulation has additional advantage.

Of course, from the viewpoint of fluid mechanics, several important assumptions are made in 1D analysis (Sherwin et al. 2003, Formaggia et al., 2003). It only solves the axial distributions of the velocity and concentration fields, and their profiles within the cross section are assumed to be fixed, e.g., parabolic for a Newtonian fluid. In addition, the flow resistance at a branching point is neglected. However, it is still unknown to what extent the errors caused by the above-mentioned assumptions would affect the present results quantitatively when 1D analysis is used instead of 3D analysis. Another important feature of the current 3D analysis is that it could be extended to analyze the dynamical coupling between red blood cells and plasma flow, and also the diffusion and consumption of oxygen within the tissue in future study. The above discussions are now added in the second paragraph of Sec. 5 (Discussion) in the revised manuscript.

[ref]

- S. Sherwin, V. Franke, J. Peiro, K. Parker “One-dimensional modelling of a vascular network in space-time variables”, J. Eng. Math. Vol. 47, No.3-4, 217-250 (2003)
- L. Formaggia, D. Lamponi, A. Quarteroni, “One-dimensional models for blood flow in arteries”, J. Eng. Math. Vol. 47 No. 3-4, 251–276 (2003).

Comment 2-6: Also, describe the calculation of Q. Looking at the velocity graphs, there is a four-order-of-magnitude variation in velocity graphed, and it looks like many values at the lower end are really stagnant flow tubes. Do these unbalance the calculation? They should not play a role in overall blood flow.

[Answer]

Actually, the flow intensity of the angiogenic front strongly depends on a sample. As shown in Figs. 2E and F, relatively large magnitudes of blood velocity can be confirmed even in the angiogenic front for *Prkci* knock-out mouse, while the blood velocity in the angiogenic front is drastically suppressed for the highly branched network in a *Foxo1* knock-out mouse. The current simulation does not allow the interstitial flow (the flow velocity is exactly zero inside the tissue), so that all the blood flow introduced from the inlet artery should flow through the vessel region, return to the vein and exit from the outlet. Therefore, it is reasonable that there is almost no blood flow at the very end of the angiogenic front. However, it does not mean there is no effect of the blood flow at the angiogenic front. Considering the molecular diffusion coefficient of the oxygen, the typical time-scale needed for oxygen to be diffused within a distance of 100 micrometer is in the order of ten seconds. Therefore, if there exists blood flow within a certain distance, oxygen can be supplied to the tissue through the combination of blood flow and molecular diffusion. We can also estimate the significance of the convection by the Peclet number, which is the ratio of the convection and diffusion effects. The Peclet number is around unity even when the blood velocity is 1% of the inlet velocity. This means that the blood transport has a similar impact to the molecular diffusion even when the blood velocity is around 1% of the inlet velocity. In conclusion, even though the blood velocity is relatively small around the angiogenic front, it could have a considerable contribution to oxygen transport. The above discussions have been added in the third to the last paragraph of Sec. 3 of the revised manuscript. We also revised the caption of Fig. S2 to clearly state that the color contours are presented in a logarithmic scale.

Comment 2-7: It would also be helpful to be able to see the code shared for the various steps here, especially since a different method is being used than is typical.

[Answer] Our codes are all in-house codes and written in Fortran language. Unfortunately, they are not written for anyone to read and run immediately. We will consider making these codes available to the public after preparing supplementary user manuals. It requires substantial time and efforts, and therefore remains as future work.

Comment 2-8: If the azimuthal flow is reduced, then the blood needs an alternate path to get from artery to vein. It looks like (Fig 2E, F) this is probably happening via large (or larger) diameter shunts in the inner network. To what extent is the lower/higher flow in the periphery a result of different network architecture in the periphery vs. different network architecture in the inner network?

[Answer] We appreciate the reviewer’s insightful comment. In order to answer the question, we revisited our simulation results. Specifically, in the original manuscript, we divide the entire vascular bed into the inner (0-70%) and outer (70-100 %) regions. We further divide the inner region into two regions, so that the entire vasculature is now divided into three regions, i.e., inner (0-30%), middle (30-70%) and outer (70-100%) regions (see Fig. R2 A attached to this reply).

Figure. R2 B and C show the changes in the azimuthal flow rates within the three regions for the network from *Foxo1* and *Prkci* KO mice. The red and blue bars represent mutant and control cases, respectively. As already discussed in the original manuscript, the flow rate in the outer region decreases for *Foxo1* KO mice (hyper-branching) and increases for *Prkci* KO mice (hypo-branching) networks, respectively. Since we divided the original inner region into two, more detailed information can be obtained. Specifically, in the case of *Foxo1* KO mice, even though the network evenly becomes dense within the entire vasculature, the flow rate is selectively increased in the immediate inner region (0-30%), while the flow rate already starts decreasing in the middle region (30-70%). This indicates that the inner shunt close to the central region has a significant impact on the downstream flow distribution. In contrast, in the case of *Prkci* KO, the flow rates in both the inner and middle regions are reduced due to hypo-branching, and the corresponding increase in the flow rate occurs in the outer region. It would be interesting to divide the entire vasculature into further narrower regions for more detailed investigation of local contributions, but it increases variations due to the lack of the number of samples. We have added above discussions on the impact of inner shunts in the last paragraph of Sec. 5 (Discussion) and also newly added Sec 6.3 and Fig. S5 (Supplementary Information) in the revised manuscript.

Figure R2. A) Decomposition of the entire vasculature into inner (0-30%), middle (30-70%) and outer (70-100%) regions for WT and KO mice. Comparison of the azimuthal flow rates in the three regions for B)*Foxo1* KO mice and (C) *Prkci* KO mice with their control littermates are shown, respectively.

Comment 2-9: Related to this, the paper claims that hyperbranching in the periphery is leading to the lower flow, leading to VEGF increase (from the abstract: "hyper-branching morphology attenuates effective blood flow at the angiogenic front and promotes tissue hypoxia”); I think it *much more likely* that inner shunts lead to lower peripheral flow, leading to VEGF increase, leading to hyperbranching. It seems much more likely to be this order, and matches with our understanding of other retinal syndromes, e.g. ROP. How to tell the difference between these causal orderings? I can think of at least one possibility: hybrid simulations with the inner network of one mouse type and the peripheral network of a different mouse type (e.g. inner control/periphery Foxo1iDEC, and vice versa). I think you also need to consider the impact of the artery and vein architecture themselves - e.g. narrowing of the artery as it moves peripherally would fav or shunting.

[Answer] Thank you for your critical comment to improve our manuscript. First of all, we do not mean “hyperbranching in the periphery is leading to the lower flow”. Specifically, when we say “hyper-branching”, it means that the vessel complexity, density and the number of branching points are uniformly increased within the entire vascular network.

In order to clarify which region has a significant impact on the blood flow distribution, the proposed hybrid simulation is ideally interesting. In practice, however, it is not straightforward since each sample have different geometry and the locations of artery and vein do not match exactly. Therefore, connecting the inner and peripheral regions from different samples is unfeasible. We could artificially introduce inner shunts or enlarge/shrink one of vessels in the current samples, but there are a lot of possibilities for achieving them so that the results will always be subjective. Because of the above reasons, we decided not to conduct additional simulations with hybrid or artificial structures. Instead, the additional results of flow modifications as shown in Fig. R2 of this reply would be useful. As the reviewer speculated, the inner shunts closer to the central part have larger impact on the mitigation of the flow rate in the angiogenic front. These results and discussions have been added in Supplementary Information Sec. 6.3 together with a newly inserted Fig. S5 in the revised manuscript.

From the biological viewpoints, during vessel growth, ECs are actively proliferating just behind the angiogenic front and around the vein, but not around the artery (M. Ehling et al., 2013). Then, ECs migrate toward upstream of the flow and integrated into the artery (M. E. Pitulescu et al., 2017). c-Myc is one of the major power generators of endothelial proliferation (Wilhelm et al. 2016). Previous reports indicate that EC proliferation of *Prkci* EC specific inducible KO mice results in reduced EC proliferation via compromised c-Myc expression (Riddell et al. 2018), while Foxo1 EC specific inducible KO exhibit enhanced c-Myc expression and increased EC proliferation in both angiogenic front and vein (Wilhelm et al. 2016, Riddell et al. 2018). As reviewer’s pointed out, to address the effects of inner shunt formation or arterial/venous formation would be very interesting. For instance, *Pard3* EC specific inducible KO mice shows reduced vessel branching and increased vessel pruning, although c-Myc expression is not affected (Hikita et al. 2018). Additionally, EC migration toward blood flow is compromised. Another interesting example is EC specific integrin b1 KO mice, which showed attenuated EC migration (Yamamoto et al., 2015). To apply our proposed numerical approach to those two models would be interesting. We included the above discussions in the last paragraph of Sec. 5 (Discussion) in the revised manuscript.

[Ref]

- E. Manuel et al., “Notch controls retinal blood vessel maturation and quiescence”, Development 140, 3051-3061, 2013.
- M. E. Pitulescu et al., “Dll4 and Notch signalling coules sprouting angiogenesis and artery formation”, Nat Cell Biol., Vol. 19, No.8, 2017.
- K. Wilhelm et al., “FOXO1 couples metabolic activity and growth state in the vascular endothelium,” Nature, vol. 529, no. 7585, p. 216, 2016.
- M. Riddell et al., “aPKC controls endothelial growth by modulating c-Myc via FoxO1 DNA-binding ability,” Nat. Commun., vol. 9, no. 1, p. 5357, 2018.
- T. Hikita et al., “PAR‐3 controls endothelial planar polarity and vascular inflammation under laminar flow” EMBO Rep., e45253, 2018
- H. Yamamoto et al., “Integrin b1 controls VE-cadherin localization and blood vessel stability”, Nat. Commun., Vol.10, no. 10, p. 6429, 2015.

Comment 2-10: While the raw intensity numbers in the region of interest at the angiogenic front seem different among the various cases, I think some normalization may be in order. for example, it looks like intensity overall in the Foxo1iDEC case is higher throughout the retina, and intensity throughout is lower in the PrkciiDEC case. Given that the conclusion is about the angiogenic front, it would seem reasonable to also compare other regions of interest, e.g. a more central region away from the front, to see whether the flow there (which is more similar among the cases) correlates with local VEGF production.

[Answer] We apologize for the insufficient description. Since the experiments with each of the hyper- and hypo-branching mutant groups were carried out independently, the basal signal intensity detected for VEGF-A was different across the mutant groups. Therefore, the mean gray value of the VEGF-A signal at the angiogenic front was normalized by the average mean gray values read from the regions showing background noise, e.g. the torn parts of the tissue or the optic nerve head hole in the middle. The above explanations have been added in the second paragraph of Sec. 4 (Experimental Validation).

Minor

Comment 2-11: Caption to Fig 3 notes n>=3; Fig 4 notes n>=4; but table 1 lists all models and it looks like n=3 for each group (same is noted in the methods section). Please confirm and use explicit n values for each group.

[Answer] We apologize for confusing descriptions in the figure captions. We assessed, at least, three control (wildtype ‘WT’) and three knock-out (KO) cases in all the experiments. Specifically, the flow simulation was only carried out for three WT and three KO animals in both of the hyper- and hypo- groups (n = 3). On the other hand, the vegf expression assessment was carried out with four samples. Accordingly, we changed from “n>=3” to “n = 3” in the caption of Fig. 3, while “n>=4” to “n = 4” in the caption of Fig. 4. Additionally, we added “n = 5 for D and G, n = 4 for E, F, H and I” into the caption of Fig.1.

Comment 2-12: Fig 3E, 3F are the same networks as Fig 2E, 2F. It would be interesting to see some of the other retinal networks that were simulated.

[Answer] We simulated three sets of WT and KO retinas per group (hyper- and hypo-branched morphologies). Following the reviewer’s comment, we have added two more supplementary figures to the manuscript (Figure S3 and S4) including simulation results the other two sets of cases from each group. Also, Figs. S3 and S4 are referred to in the first paragraph of Sec 3 in the revised manuscript.

Comment 2-13: Is there a reason only one flow region (wedge) was chosen for each mouse? How was this region chosen, both in terms of its size and location? Multiple regions per mouse would improve confidence in the predictions.

[Answer] The flow analyses were carried out only in a subset of the retinal vasculature containing one arterial- and venous-branch and the capillary bed in between. This specific subset of the vasculature was chosen as the experimental unit for the morphometric measurements and flow analyses because of the following difficulties and limitations

1- Containing one artery (inlet), one vein (outlet) and the network of the capillaries in between, the chosen subset can be considered as a one complete flow transportation unit.
2- Due to the hemispherical geometry of the retinal tissue, radial cuts are needed in order to facilitate the flat mounting of the tissue for imaging. With the four cuts dividing the retinal tissue into four flat lobes, the retinal vascular network is damaged. Also, some parts of the retinal tissue would be damaged during the staining processes. Consequently, it was difficult to find a whole region containing intact connections of artery, vein and all the capillaries, from the optic nerve head to the angiogenic front.
3- To lower the computation costs, we tried to work with the minimum subset that can be considered as one complete transportation unit. The validity of considering the minimum flow unit has also been proved in our reply to the reviewer’s comment 2-3.

The above explanations and the discussions on the validity of the present simulation have been added in Sec. 6.1. (Supplementary Information) in the revised manuscript.

**[Others to the Reviewers]**

Have all data under lying the figures and results presented in the manuscript been provided? Large-scale datasets should be made available via a public repository as described in the PLOS Computational Biology data availability policy, and numerical data that underlies graphs or summary statistics should be provided in spreadsheet form as supporting information.

Reviewer #1: None

Reviewer # 2: No: Code is not provided; digitized networks not provided; images for most retinas/simulated networks not provided

[Answer] Due to the complexity of the in-house Fortran codes used in the present study, making them available to the public will be consider in the future after developing necessary documents such as user manuals etc. The simulation results of all three samples for the networks from *Foxo1* and *Prkci* KO mice considered in the present study have been added as the supplementary Figs. S3 and S4. In the acceptance of the present manuscript, all digital data required to reproduce the results and plots included in the present manuscript will be made available via a public repository in accordance with the PLOS Computational Biology data availability policy.

PLOS authors have the option to publish the peer review history of their article (what does this mean?). If published, this will include your full peer review and any attached files.

If you choose “< span lang=EN-US>no”, your identity will remain anonymous but your review may still be made public.

Do you want your identity to be public for this peer review? For information about this choice, including consent withdraw al, please see our Privacy Policy.

Reviewer #1: No

Reviewer #2: No
