# Supplementary material for: Numerical evaluation reveals the effect of branching morphology on vessel transport properties during angiogenesis
Source: PLoS Comput Biol. 2021 Jun 16;17(6):e1008398. doi: 10.1371/journal.pcbi.1008398 (PMC8238234; doi:10.1371/journal.pcbi.1008398)
Supplement: Attachment — Submitted filename: point by point .docx [file pcbi.1008398.s012.docx]

**Reviewer #1:** My concerns have been adequately addressed

**Answer**: we would like to thank the reviewer for his/her time and helpful comments.

**Reviewer #2:** The authors have done a good job replying to the comments and critiques overall. While there are some additional simulations I'd love to have seen for scientific interest reasons, I don't think that their absence stands in the way of publication.

**Answer:** we would like to thank the reviewer for his/her time and helpful comments.

**1. The simulation code should be shared. While commenting and documentation are best practice, their absence should not stand in the way of sharing the code. This would aid review and facilitate reproducibility. It is absolutely standard in experimental sciences that experimental reagents are shared, and codes and models are essentially our reagents.**

**Answer:** as suggested we uploaded the simulation code on the server.

**2. In the abstract, the following statement still seems not fully supported:

"Interestingly, hyper-branching morphology attenuates effective blood flow at the
angiogenic front and promotes tissue hypoxia."

I accept the authors' argument that in the critique I over-interpreted this as claiming that hyperbranching *in the periphery* was reducing flow - rather than the hyperbranched network overall - and the expanded discussion of the impact of inner & outer network regions helps to clarify this. I still think that this sentence in the abstract claims tissue hypoxia effects that haven't been measured here (though highly likely) - and which of course could result in further branching. Perhaps '...attenuates effective blood flow at the angiogenic front, likely promoting tissue hypoxia'?**

**Answer:** accordingly, we collected our description in the abstract, page2 line 32-33.

Interestingly, hyper-branching morphology attenuates effective blood flow at the angiogenic front, likely promoting tissue hypoxia.
